# Validation of the FluoroType MTBDR Assay for Detection of Rifampin and Isoniazid Resistance in Mycobacterium tuberculosis Complex Isolates

**DOI:** 10.1128/JCM.00072-18

**Published:** 2018-05-25

**Authors:** Doris Hillemann, Carsten Haasis, Sönke Andres, Tobias Behn, Katharina Kranzer

**Affiliations:** a National Reference Laboratory, Research Centre Borstel, Borstel, Germany; b London School of Hygiene and Tropical Medicine, London, United Kingdom; Carter BloodCare & Baylor University Medical Center

**Keywords:** tuberculosis, molecular methods, isoniazid, rifampicin, drug resistance, molecular diagnostics

## Abstract

For Mycobacterium tuberculosis complex (MTBC), the rapid and accurate diagnosis of drug resistance is crucial to ensure early initiation of appropriate therapy. Recently, a new molecular diagnostic test, the FluoroType MTBDR, aimed at detecting rifampin and isoniazid resistance has become available. This study aimed to evaluate the FluoroType MTBDR in comparison to phenotypic drug susceptibility testing (DST) using M. tuberculosis complex isolates. MTBC isolates underwent phenotypic DST and were tested using the FluoroType MTBDR and Genotype MTBDRplus. Sanger sequencing of the key regions of *rpoB*, *katG*, *inhA*, and *aphC* was performed for isolates with discordant phenotypic and molecular results. Furthermore, isolates with specific wild-type bands missing in the Genotype MTBDRplus, indicating the presence of a mutation, were investigated by Sanger sequencing. Specificity and sensitivity, defined as the proportions of isolates correctly determined as susceptible and resistant by the FluoroType MTBDR compared to phenotypic DST, were calculated. A total of 180 culture isolates were included; phenotypic DST showed 85 isolates susceptible to isoniazid and rifampin, 7 with isoniazid monoresistance, 7 with rifampin monoresistance, and 81 with multidrug resistance. The specificity of the FluoroType MTBDR was 100% (95% confidence interval [CI], 96.0 to 100%) for both rifampin and isoniazid. The sensitivity was 91.7% (95% CI, 83.6 to 96.6%) for isoniazid and 98.9% (95% CI, 93.8 to 100.0%) for rifampin. The FluoroType MTBDR has a high sensitivity and specificity for the detection of rifampin and isoniazid resistance when using culture isolates.

## INTRODUCTION

Rapid and accurate laboratory diagnoses of Mycobacterium tuberculosis drug susceptibility and resistance are crucial to ensure early initiation of appropriate therapy, to adequately manage disease, and to control further transmission. Conventional drug susceptibility testing (DST) relies on culture-based methods with results available only after several weeks. Much shorter turnaround times can be achieved with molecular diagnostics performed either directly on sputum samples or on positive cultures (1–3).

The currently available molecular methods include Xpert MTB/RIF, line probe assays (LPAs), Sanger target sequencing, and next-generation sequencing (4–7). LPAs are recommended by the World Health Organization (WHO) as rapid diagnostic tests for detection of drug resistance (8). The technology combines PCR with subsequent reverse hybridization. The specifically bound amplicons are made visible in a colorimetric detection reaction resulting in banding patterns suggestive of the presence or absence of mutations in the target genes. The most widely used LPA, the GenoType MTBDRplus, detects the most prevalent mutations in the *rpoB*, *katG*, *inhA* genes conferring resistance to rifampin (RMP) and isoniazid (INH). The Genotype MTBDRplus is designed to specifically detect four *rpoB* mutations (in codons 516, 526, and 531), two *katG* mutations (in codon 315), and four *inhA* mutations in the regulatory region. Other mutations within the amplified region of the target genes are indicated by the absence of a wild-type band without the simultaneous presence of a mutation band.

Recently a new assay, the FluoroType MTBDR, which is directed at the same target genes but uses a different technology, has been developed. This assay combines a linear-after-the-exponential PCR (LATE-PCR) (9) with special probes using lights-on/lights-off detection technology (10). The readouts of the FluoroType MTBDR are melting curves. The shapes correspond to wild types or to the presence of specific mutations. The FluoroType MTBDR is an open system that identifies characterized mutations via a learning software interpreting the melting curves.

Practically, the FluoroType MTBDR has several advantages over the Genotype MTBDRplus: (i) less hands-on time, (ii) more-rapid results, (iii) decreased risk of DNA contamination, and (iv) automatic interpretation with the possibility to import results directly into a laboratory information system. However, the sensitivity and specificity of this new method for detecting RMP and INH resistance have not been investigated under routine conditions.

This study aimed to compare the results of phenotypic DST, GenoType MTBDRplus, and FluoroType MTBDR using cultures positive for M. tuberculosis complex (MTBC).

## MATERIALS AND METHODS

Mycobacterial cultures were performed at the National Mycobacterial Reference Laboratory in Borstel, Germany, between May 2016 and October 2017 using mycobacterial growth indicator tubes (MGIT) (Becton Dickinson, USA) and Loewenstein-Jensen and Stonebrink culture slants (Artelt-Enclit, Germany). All cultures were identified to species level using the GenoType MTBC version 1.0 (Hain Lifescience, Nehren, Germany) according to the manufacturer's instructions. The MGIT 960 isoniazid-rifampin-ethambutol (IRE) kit (Becton Dickinson) was used according to the manufacturer's instructions. The critical concentrations of rifampin and isoniazid were 1 μg/ml and 0.1 μg/ml, respectively (11).

For DNA extraction, an aliquot of 500 μl was obtained from the bottom of a positive MGIT or two loops (10 μl) of bacterial growth were collected from Loewenstein-Jensen or Stonebrink culture slants and added to 500 μl of 0.9% NaCl solution. Samples were centrifuged at 3,000 × *g* for 15 min. Supernatants were removed, and DNA was extracted using the Fluorolyse kit (Hain Lifescience, Nehren, Germany) according to the manufacturer's instructions. In brief, 100 μl of lysis buffer and 2 μl of internal control were added to the remaining pellets. The mixed samples were incubated at 95°C for 5 min, centrifuged for 1 min, and resuspended in 100 μl neutralization buffer. Following centrifugation at 10,000 × *g* for 5 min, supernatants were transferred into a 0.5-ml cryovial. DNA was stored at −20°C. For each batch, one negative control using 100 μl of lysis buffer, 100 μl of neutralization buffer, and 2 μl of internal control was included.

DNA was analyzed with the Genotype MTBDRplus version 2 and FluoroType MTBDR assays (Hain Lifescience, Nehren, Germany) as per the manufacturer's instructions. In brief, for the FluoroType MTBDR, 20 μl of DNA solution was added to 6 μl master mix component A and 14 μl master mix component B and transferred into 96-well plates. The plates were then sealed, centrifuged, and analyzed via FluoroCycler 96. For each plate, one positive and one negative control were included. For the Genotype MTBDRplus version 2, 5 μl of DNA solution was added to 45 μl of master mix containing 10 μl master mix component A and 35 μl master mix component B. Twenty microliters of PCR products was added to 20 μl denaturation reagent in a hybridization well. Hybridization reagents were prepared, preheated, and loaded onto the GT-Blot48 (Hain Lifescience, Nehren, Germany). After an initial hybridization step, strips were added and hybridization performed as per the standard protocol. For each batch, one negative control (45 μl master mix, 5 μl DNA-free water) was tested. Interpretation of the results was performed according to the manufacturer's instructions.

The package insert of the FluoroType MTBDR identifies the following *rpoB*, *katG*, and *inhA* mutations in amino acid codons (e.g., S531L) and regulatory regions (e.g., T-8A): T508A, S509T, E510H, L511P, S512K, Q513L, Q513P, Q513R, D516A, D516F, D516V, D516Y, N518I, S522L, S522Q, H526C, H526D, H526G, H526L, H526N, H526P, H526Q, H526R, H526S, H526Y, R529K, S531F, S531L, S531L, S531Q, S531W, L533E, L533P, S315T1, S315T2, S315N, S315R, G-17T, A-16G, C-15T, G-9A, T-8A, T-8C, and T-8G. In contrast the Genotype MTBDRplus is able to detect and specify the following mutations: D516V, H526Y, H526D, S531L, S315T1, S315T2, C-15T, A-16G, T-8C, and T-8A.

Molecular results were interpreted without knowledge of phenotypic DST results. Sanger sequencing in the key regions of *rpoB*, *katG*, *inhA*, and *aphC* (12) was performed for isolates with discordant results between either the two molecular methods or the molecular and phenotypic methods. If the Genotype MTBDRplus showed a missing wild-type band but not a mutation band, Sanger sequencing in the respective key region was performed to confirm the FluoroType MTBDR result. Sanger sequencing was performed using an ABI 3130xl genetic analyzer (Applied Biosystems) and an ABI BigDye Terminator cycle sequencing kit (version 3.1) according to the manufacturer's instructions.

Molecular results were coded as wild type, individual mutation, mutations in a region of the gene, or indeterminate. The molecular results were interpreted as susceptible, resistant, or indeterminate. Indeterminate results in the Genotype MTBDRplus were defined as detection of MTBC DNA without the presence of a gene locus control. All samples yielding an indeterminate result were retested using a new aliquot of DNA. A mixed culture (heteroresistance) was defined as both wild-type and mutant DNA being present as identified by wild-type and mutant bands (Genotype MTBDRplus) and/or by respective double peaks in the DNA sequence.

To determine the accuracy of the FluoroType MTBDR assay, the results were compared with phenotypic DST results. Sensitivity and specificity were calculated for RMP and INH separately. Sensitivity was defined as the proportion of isolates correctly determined as resistant by the FluoroType MTBDR compared to the phenotypic DST results. Specificity was defined as the proportion of isolates correctly determined as susceptible by the FluoroType MTBDR compared to the phenotypic DST results. The McNemar test on paired proportions was used to compare the sensitivities of the FluoroType MTBDR and the Genotype MTBDRplus for INH. All statistical analysis were performed using Stata 14.0.

Information was extracted from the laboratory information system anonymously. The ethics committee of the University of Luebeck approved the study. Individual consent was not sought as no additional patient information was obtained.

## RESULTS

A total of 180 cultures were included in the study. The majority were identified as M. tuberculosis (*n* = 173), followed by Mycobacterium africanum (*n* = 5), Mycobacterium bovis subsp. *bovis* (*n* = 1), and M. bovis BCG (*n* = 1). Analysis of RMP and INH resistance included all isolates regardless of species. Eighty-five isolates tested phenotypically susceptible for both RMP and INH, 7 were INH monoresistant, 7 were RMP monoresistant, and 81 were resistant to both RMP and INH (Table 1). The number of indeterminate results by FluoroType MTBDR was 1 (0.6%) for *rpoB*, 2 (1.1%) for *inhA*, and 6 for *katG* (3.3%) initially. Following repeated DNA extraction, the number of indeterminate results was 0 (0.0%) for *rpoB*, 1 (0.6%) for *inhA*, and 3 for *katG* (1.7%). Sensitivity of the FluoroType MTBDR for INH was 91.7% (77/84; 95% confidence interval [CI], 83.6 to 96.6%), and specificity was 100% (92/92; 95% CI, 96.1 to 100.0%). For RMP, the sensitivity was 98.9% (87/88; 95% CI, 93.8 to 100.0%), and the specificity was 100.0% (90/90; 95% CI, 96.0 to 100.0%). The most frequent *rpoB* mutation detected by FluoroType MTBDR was S531L (*n* = 56). A total of 7 *rpoB* mutations remained unidentified. Mutations were identified by Sanger *rpoB* sequencing as H526L (*n* = 3), S531L (*n* = 1), Q513K (*n* = 1), D516Y (*n* = 1), and, in one mixed culture, H526D and S531L. A total of four mixed culture were identified in the study. One of those cultures was a mixture of an *rpoB* wild-type isolate and an isolate with a L533P mutation. The FluoroType MTBDR detected the L533P mutations, while the Genotype MTBDRplus did not. The FluoroType MTBDR identified three isolates as having H526P mutations, while Sanger sequencing revealed H526L mutations. The FluoroType MTBDR falsely detected an H526R mutation in a mixed culture with one isolate harboring an H526D mutation and the other a S531L mutation. Of the seven cultures falsely tested as INH susceptible by the FluoroType MTBDR, four had *katG* and/or *inhA* mutations detected by the Genotype MTBDRplus. This resulted in a higher, but not significantly, sensitivity of the MTBDRplus (96.0%; 95% CI, 89.9 to 99.3%) for INH than of the FluoroType MTBDR (91.7%; 95% CI, 83.6 to 96.6%) (*P* = 0.12).

**TABLE 1:** Results with the FluoroType MTBDR, GenoType MTBDRplus, and phenotypic DST for all 180 isolates^*a*^

No. of isolates	FluoroType MTBDR					GenoType MTBDRplus^*b*^					Phenotypic DST		Sanger sequencing			
	*rpoB*	*katG*	*inhA*	Molecular DST interpretation		*rpoB*	*katG*	*inhA*	Molecular DST interpretation							
				RMP	INH				RMP	INH	RMP	INH	*rpoB*	*katG*	*inhA*	*alpC*
85^*d*^	WT	WT	WT	S	S	WT	WT	WT	S	S	S	S				
6	WT	S315T1	WT	S	R	WT	S315T1	WT	S	R	S	R				
1	WT	WT	C-15T	S	R	WT	WT	C-15T	S	R	S	R				
1	WT	S315T1	C-15T	S	R	WT	S315T1	C-15T	S	R	R	R	Mutation outside the hot spot region			
3	S531L	WT	WT	R	S	S531L	WT	WT	R	S	R	S				
1	S531L	WT	WT	R	S	S531L	WT	WT	R	S	R	R		WT	WT	WT
3	S531L	WT	WT	R	S	530–533	WT	WT	R	S	R	S	S531L			
1	S531L	WT	WT	R	S	530–533	WT	WT	R	S	R	R	S531L	WT	WT	C-10T
1	S531L	WT	WT	R	S	S531L	WT	T-8A	R	R	R	R			T-8A	
1	S531L	IND	WT	R	IND	S531L	IND	WT	R	IND	R	R		WT	WT	WT
1	S531L	MUT	WT	R	R	S531L	S315T1	WT	R	R	R	R		S315T1		
2	S531L	S315T2	WT	R	R	S531L	S315T2	WT	R	R	R	R				
2	S531L	S315T1	C-15T	R	R	S531L	S315T1	C-15T	R	R	R	R				
1	S531L	S315T1	C-15T	R	R	530–533	S315T1	C-15T	R	R	R	R	S531L			
1	S531L	S315T1	T-8C	R	R	S531L	S315T1	T-8C	R	R	R	R				
38	S531L	S315T1	WT	R	R	S531L	S315T1	WT	R	R	R	R				
1	S531L	S315T1	WT	R	R	530–533	S315T1	WT	R	R	R	R	S531L			
5	D516V	S315T1	WT	R	R	D516V	S315T1	WT	R	R	R	R				
1	D516V	S315T1	WT	R	R	513–519	S315T1	WT	R	R	R	R	D516V			
1^*c*^	D516Y	WT	IND	R	IND	513–519 S531L	IND	C-15T	R	R	R	R	D516Y S531L	WT	C-15T	
1^*e*^	D516Y	WT	WT	R	S	513–519	S315T1	−15 to −17	R	R	R	R	D516Y	S315T1	C-17T	
3^*e*^	H526D	S315T1	WT	R	R	H526D	S315T1	WT	R	R	R	R				
1	H526D	WT	C-15T	R	R	H526D	WT	C-15T	R	R	R	R				
1	H526D	WT	WT	R	S	H526D	WT	WT	R	S	R	S				
1	H526N	S315T1	WT	R	R	526–529	S315T1	WT	R	R	R	R	H526N			
3	H526P	S315T1	C-15T	R	R	526–529	S315T1	C-15T	R	R	R	R	H526L			
1^*c*^	H526R	IND	WT	R	IND	H526D S531L	S315T1	WT	R	R	R	R	H526D S531L	S315T1		
1	H526Y	IND	WT	R	IND	H526Y	S315T1	WT	R	R	R	R		S315T1		
2^*e*^	H526Y	S315T1	WT	R	R	H526Y	S315T1	WT	R	R	R	R				
1	H526Y	WT	WT	R	S	H526Y	S315T1	WT	R	R	R	R		S315T1		
1	L533P	S315T1	T-8C	R	R	530–533	S315T1	T-8C	R	R	R	R	L533P			
1^*c*^	L533P	S315T1	WT	R	R	WT	S315T1	WT	S	R	R	R	WT L533P			
3^*e*^	MUT	S315T1	WT	R	R	526–529	S315T1	WT	R	R	R	R	H526L			
1	MUT	S315T1	WT	R	R	530–533	S315T1	WT	R	R	R	R	S531L			
1	MUT	S315T1	WT	R	R	510–517	S315T1	WT	R	R	R	R	Q513K			
1^*c*^	MUT	WT	WT	R	S	H526D S531L	WT	WT	R	S	R	R	H526D S531L	WT	WT	WT
1^*e*^	MUT	WT	WT	R	S	513–519	S315T1	−15 to −17	R	R	R	R	D516Y	S315T1	C-17T	

a Abbreviations: DST, drug susceptibility testing; RMP, rifampin; INH, isoniazid; WT, wild type; S, susceptible; R, resistant; IND, indeterminate; MUT, unidentified mutation.

b When wild-type bands were missing in the Genotype MTBDRplus assay, the base pair region is indicated (e.g., 526–529).

c Mixed cultures.

d One M. bovis subsp. *bovis* isolate and one M. bovis BCG isolate are included.

e One M africanum isolate is included.

## DISCUSSION

This study shows high sensitivity and specificity of the FluoroType MTBDR for the detection of RMP and INH resistance using M. tuberculosis complex isolates. The proportion of indeterminate results was small. Repeat DNA extraction resolved 5 of 9 indeterminate results, suggesting that insufficient or low-quality DNA might have been the problem. Two of the isolates that tested repeatedly indeterminate were mixed cultures, which might explain the result.

For RMP, the sensitivity and specificity of FluoroType MTBDR were comparable to those reported for established molecular diagnostics such as the Genotype MTBDRplus and the Xpert MTB/RIF (2, 3, 13). While the presence or absence of an *rpoB* mutation was correctly detected in all isolates, the FluoroType MTBDR failed to identify the exact mutation in 10% (9/87) of cases. Given that silent *rpoB* mutations are rare (14–16), one might argue that detecting the presence of an *rpoB* mutation itself is sufficient. In fact, the most widely used molecular diagnostic test, the Xpert MTB/RIF, indicates only the presence of RMP resistance without information about the associated *rpoB* mutation.

The sensitivity of the FluoroType MTBDR for detecting *katG* and/or *inhA* mutations was lower than that of the Genotype MTBDRplus. Two of the isolates falsely reported as INH susceptible by the FluoroType MTBDR had a rare combination of mutations, an S315T1 *katG* mutation and a C-17T mutation in the *inhA* promoter region, and one isolate had a T-8A mutation in the *inhA* promoter region. Theoretically the FluoroType MTBDR has the potential for continuous improvement when new mutations and their respective melting curve properties are detected and fed back into the analytic software. Whether and how frequently improvement through software updates will occur remain to be seen.

WHO recommends that commercial molecular LPAs such as the Genotype MTBDRplus may be used for a positive sputum smear specimen or a cultured isolate of M. tuberculosis complex to detect resistance to RMP and INH (8). The Genotype MTBDRplus offers benefits compared to phenotypic DST when used on cultured isolates. First, results are available within 48 h of the culture becoming positive, in contrast to the 7 to 14 days to results with culture-based DST (17). Second, these assays are not affected by the presence of contamination with bacteria, fungi, or nontuberculosis mycobacteria, as are liquid culture-based DST systems. The Genotype MTBDRplus and FluoroType MTBDR detect mutations in the same three target genes. However, there are some distinct advantages of the FluoroType MTBDR. Except for DNA extraction, it provides a fully automated, closed system with a capacity of testing 95 samples in one batch. This in turn decreases the risk of DNA contamination, which is less problematic when using DNA extracted from cultured isolates but may be a significant problem when testing primary samples. Full automation also means less hands-on time and automated interpretation of results, reducing the risk of analytic errors. Results can be directly imported into a laboratory information system, thus decreasing transcription errors.

This study has several strengths and limitations. It was conducted in a national tuberculosis reference laboratory with standardized procedures for phenotypic and molecular DST and long-standing expertise with LPA and Sanger sequencing. Interpretation of molecular results was performed blinded to the phenotypic DST results. Half of the isolates included in the study were resistant to both RMP and INH. However, the majority of isolates harbored the common S531L *rpoB* and S315T1 *katG* mutations, possibly skewing the sensitivity estimate upwards.

In summary, the FluoroType MTBDR assay testing culture isolates of M. tuberculosis complex has a high sensitivity for detecting RMP resistance but a lesser sensitivity for detection of INH resistance. It offers an alternative to the currently WHO-endorsed Genotype MTBDRplus, especially for high-throughput laboratories. However, validation studies from other settings are needed to ensure that isolates with a variety of mutations and combinations of mutations are investigated. Furthermore, studies in low- and middle-income countries with high burdens of multidrug-resistant tuberculosis (MDR-TB) should be performed to assess the performance of the new test in those settings.
